# Research on dynamic characteristics of cutter breaking frozen soil and optimal drum rotation speed of the new shaft tunneling machine

**DOI:** 10.1038/s41598-024-55935-4

**Published:** 2024-03-09

**Authors:** Xin-ming Chen, Zhi Yang, Hua-zhe Jiao, Jiang-ling Zhang, Jie Wang, Jin-yu Sun

**Affiliations:** 1 Henan Key Laboratory of Underground Engineering and Disaster Prevention and Control, Henan Polytechnic University, Jiaozuo, 454150 Henan China; 2 School of Civil Engineering, Henan Polytechnic University, Jiaozuo, 454150 China

**Keywords:** The new shaft tunneling machine, Rolling cutter, Dynamic damage mechanism, Finite element, Specific energy, Engineering, Civil engineering

## Abstract

To improve the efficiency of frozen soil excavation, the new shaft tunneling machine was developed. The new shaft tunneling machine exerts pressure on the frozen soil through the cutter under the joint action of its own gravity, the drum rotational force and the inertia force, and the frozen soil is damaged. By unique way of breaking frozen soil to improve the efficiency of frozen soil excavation, the drum rotation speed is one of the factors affecting the performance of frozen soil excavation. This article applies SolidWorks software to establish the model of cutter breaking frozen soil, takes advantage of Hyper Mesh finite element software coupled with LS-DYNA solver to acquire the regular pattern of change in the force change, frozen soil stress–strain and specific energy of cutter crushing frozen soil, etc., which analyzes the destruction of frozen soil when the drum of the new shaft tunneling machine is rotating at the speed of 25–40 rpm. Combine with field test to investigate the mechanism of cutter breaking frozen soil under the optimal drum rotation speed. The investigation results demonstrate that: when frozen soil's self-bearing capacity is lower than the force of cutter, it breaks up and detaches from the soil body, and frozen soil undergoes tensile, compressive and shear damages. For this research, it is instructive for practical engineering.

## Introduction

In the excavation of shafts constructed by freezing method, frozen soil is mainly a four-phase body composed of solid mineral particles, liquid, gas, and viscous-plastic ice inclusions^1,2^, which is greatly affected by temperature, water content, and other factors, i.e., the lower the temperature is, the greater the strength of the frozen soil of itself and the water content of the soil is not saturated, the strength of the frozen soil is increased with the increase of the water content^3^. In addition, the external loading environment also has a effect on the strength of frozen soil, i.e., the compressive and shear strength of frozen soil decreases with the prolongation of the external loading force and plastic deformation occurs leading to stress relaxation^4,5^. Due to the multiple influences that make the mechanical properties of frozen soil, frozen soil faces many difficulties during excavation.

The conventional excavation methods for frozen soil are currently available as the warming and thawing method, the blasting method, and the mechanical method^6,7^. The warming and thawing method is used to melt the frozen soil by using heat to achieve the purpose of excavation^8^. Although the effect is good, the energy consumption is high and the cost is high, and the action surface is small, which is not suitable for excavating frozen soil in depth shafts^9^, and it is generally more common in emergency projects and projects with small earth volumes^10^. The blasting method is economical and efficient, but the construction safety factor is low when blasting is carried out at the initial stage of the shaft wall support^11^. In the mechanical method of excavation, the majority of excavators are used to dig the frozen soil, which has the advantages of good safety and a small input, but the excavation efficiency is low. As the depth of well construction increases, problems such as alluvial thickness and difficulty of excavation emerge, and in the face of the requirements for vertical shaft trenching with ultra-deep, oversized sections and geological conditions^12,13^, the traditional excavation method of crushing frozen soil has low efficiency and slow construction speed^14^, which is not coordinated with today's project construction speed and non-compliant with the concept of green mine construction, and has become a bottleneck limiting the development of vertical shaft trenching^15^.

The researchers, both domestic and overseas, have researched a lot in improving the efficiency of excavating frozen soil for a long time^16^. Compared with the heating and thawing method and the explosion process, the mechanical method is more widely applicable, and there are fewer studies on the mechanism of mechanical excavation of frozen soil, so it is necessary to carry out more in-depth research on the mechanical excavation method of frozen soil. Lu et al.^17^ conducted cutting tests on frozen soil based on a chain-type narrow cutter and analyzed the variation of cutting force on frozen soil with temperature, cutting speed velocity, tool shape, and tool front angle. Li^18^ elaborated on the application of single-hook hydraulic excavators in the construction of frozen soil excavation projects, pointing out that single-hook excavators are easier to excavate broken frozen soil than conventional bucket-tooth excavators. Fedulov et al.^19^ and others have shown that power-driven impact buckets are more likely to break up soil and have a longer bucket life.

In addition, a multitude of researchers have given reasonable explanations in terms of the frozen soil damage theory. For more details, Yu et al.^20^ proposed that the difference in frozen soil strength originates from the material homogeneity and quantitatively gave the relationship between material characteristic length and homogeneity, which provides a theoretical basis for the measurement of frozen soil tensile strength and the design of frozen soil excavation. Tang et al.^21^ concluded that the progress of frozen excavation benefited from the frozen temperature, and stability conditions related to the thaw depth were established by finite element analysis of the temperature in the tunnel. Zhelnin et al.^22^ established the THM model of water-saturated soil freezing by using the porous mechanics theory of Cousse and studied the mechanical behavior of the soil. In addition, the constitutive relationship between plastic volume strain caused by frost heave and the viscoelastic strain related to the rheological properties of frozen soil were considered. The finite element method (FEM) was used to solve the problem, and the influence of frost heave and water migration on the sinking and excavation activities of the shaft was studied. Most of the above studies are limited to blasting excavation of frozen soil and cutting damage to frozen soil by tunneling machine, as well as the theoretical basis of frozen soil damage, which has improved the efficiency of frozen soil excavation to a extent, but most of the experimental studies on the mechanical properties of frozen soil are focused on static and quasi-static aspects^23^, and few studies on the dynamic damage of frozen soil under the joint action of mechanical gravity and rotational force have been involved.

Based on this, the 3D modeling software SolidWorks is used to establish a new shaft tunneling machine rolling cutter breaking frozen soil model, through the finite element analysis software HyperMesh coupled with the solver LS-DYNA^24^, to simulate the process of rolling cutter breaking frozen soil under different drum rotation speeds, and to obtain the damage condition of frozen soil under different drum rotation speeds, combined with the force change, frozen soil stress–strain change and specific energy and other rules of change, to select the optimal drum rotation speed; combined with field tests to analyze the process of cutter breaking frozen soil under the optimal drum rotation speed, to improve the rolling cutter breaking frozen soil under the optimal drum rotation speed.

## Methods

**Figure 1:**
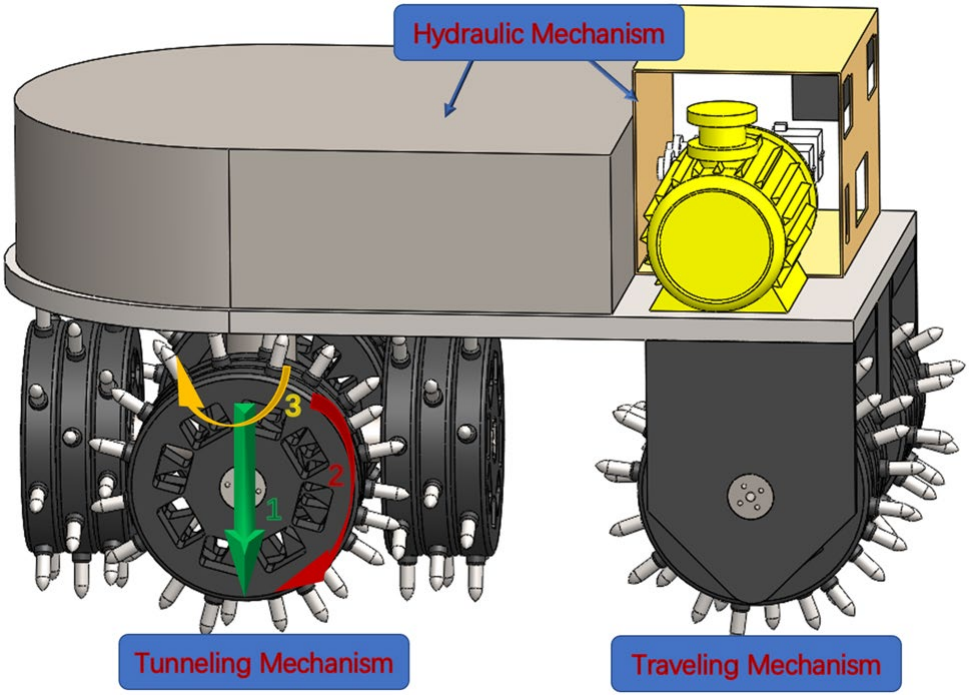
Model diagram of new shaft tunneling machine and the drum movement method.

For the sake of meeting the requirements of shaft frozen soil excavation under the conditions of ultra-deep, oversize section and low temperature, a new type of shaft tunneling machine has been developed independently, which includes a hydraulic mechanism, a tunneling mechanism and a traveling mechanism. The 3D model is shown in Fig. 1. In the process of tunneling frozen soil, the hydraulic mechanism provides power for the tunneling mechanism, and the traveling mechanism controls the advance and steering of the new shaft boring machine, and the unique way of breaking the frozen soil of the new shaft boring machine improves the efficiency of excavating frozen soil.

As shown in Fig. 1, the tunneling mechanism of the new shaft tunneling machine is composed of a vertical shaft, a cross shaft and a drum component, and a plurality of cutter are uniformly distributed on the drum. Dynamic breaking of frozen soil by a cutter is an process that can be viewed as a superposition of three forms of motion: (1) The cutter intrudes into the frozen soil under the force of the equipment's own gravity and moves in an approximately straight line in the vertical tunneling direction; (2) The cutters are evenly distributed on the drum and move in a circular motion around the drum under the rotational force of the drum; (3) The cutter moves in a circular motion on the drum around the vertical axis.

### Mechanism analysis of breaking frozen soil by cutter

In the process of tunneling, the cutter rolls along the surface of frozen soil excavation, and the rotating drum exerts pressure on each cutter, thus forming a rolling extrusion on the surface of frozen soil, and each rotation of the drum penetrates into the surface of frozen soil at a depth. During the intrusion of the cutter into the frozen soil, the frozen soil is squeezed and cracked, producing a number of cracks, starting from the region, the cracks expand in all directions, due to the fact that the bearing capacity of the frozen soil that has been cracked becomes smaller, and cannot withstand the impact force brought about by the cutter, the formation of a block of debris, and ultimately dislodged^25,26^. Since the destruction of frozen soil under the rolling action of cutter is an dynamic destruction process, which has an impact on the efficiency of breaking frozen soil^27,28^, it is of great significance to study the breaking mechanism of frozen soil under the rolling action of cutter.

**Figure 2:**
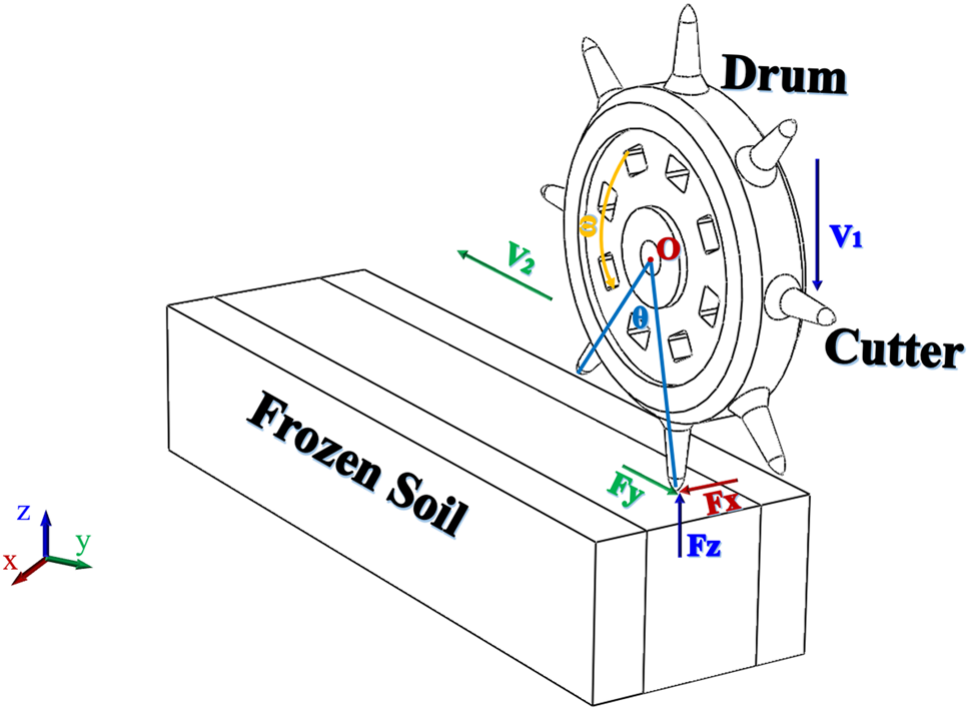
Force analysis of frozen soil based on the action of the rotating cutter.

Cutter breaking frozen soil is a gradual process, which can be divided into three stages: preliminary intrusion, local intrusion and regional breaking. The force analysis of the cutter breaking frozen soil is shown in Fig. 2, when the drum rotates around the X-axis with a rotational speed, the cutter rolls into the frozen soil with different angle θ, which is mainly subject to lateral force Fx, rolling force Fy and normal force Fz.

The combined force on the cutter is also one of the indexes for evaluating the breaking efficiency of the cutter^29,30^, and the combined force on the cutter can be calculated by Eq. (1).

1 $$F_{{}} = F_{y} + \sqrt {\left( {F_{x} } \right)^{2} + \left( {F_{{\text{z}}} } \right)^{2} }$$

The calculation of the cutter force to simplify the calculation, based on the V-shaped cutter model for force analysis, this is due to the cutter blade width is generally only a dozen millimeters, the mechanical properties of the cutter has little impact, so do not consider the impact of the cutter blade width. The depth of cutter intrusion into frozen soil is simplified as the digging depth corresponding to one revolution of the drum, which is numerically equal to the penetration.

**Figure 3:**
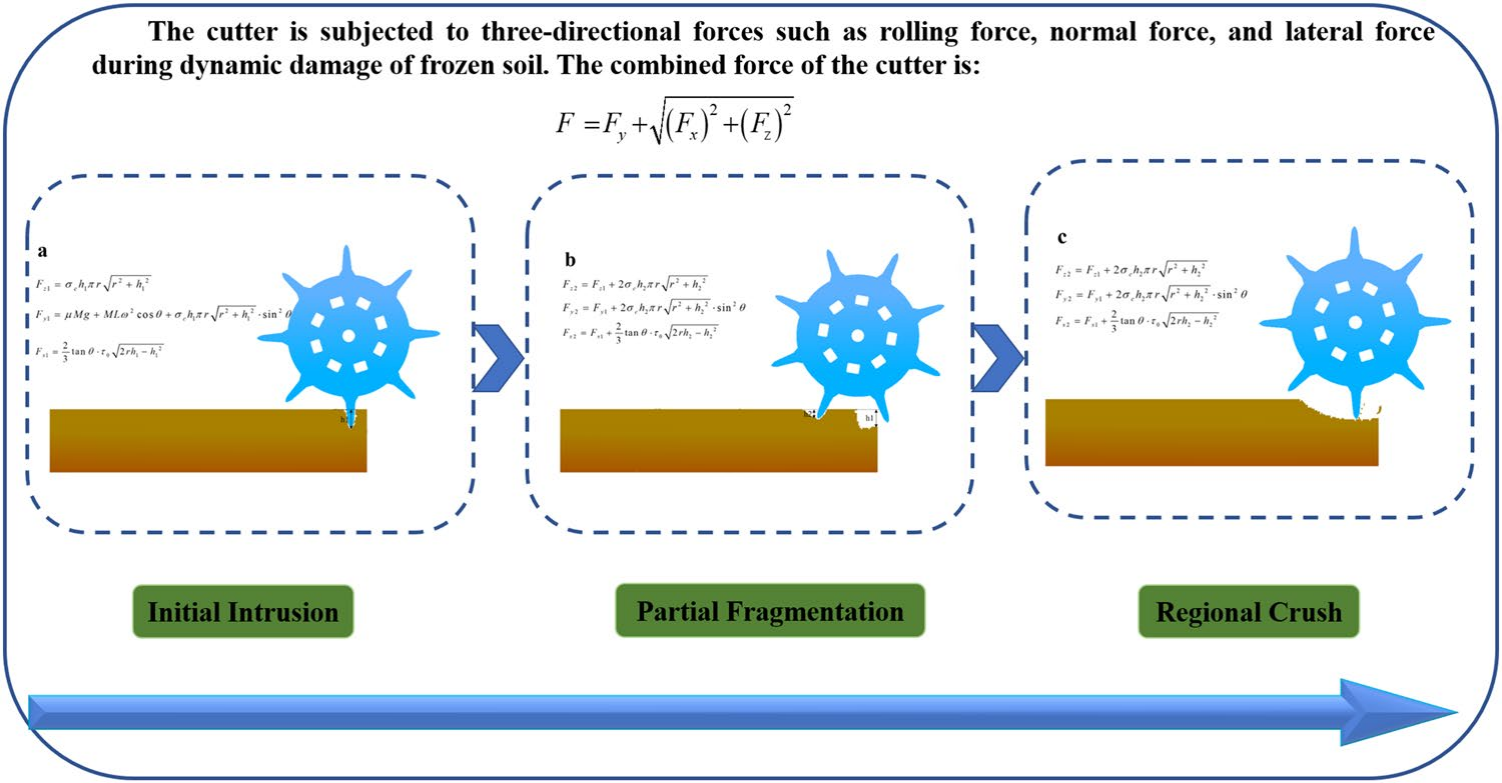
Force equations of cutter under different cutter breaking frozen soil process.

Analyze the preliminary invasion stage, as shown in Fig. 3, the cutter in the new shaft boring machine under the action of its own gravity invades a depth, with the rotation of the drum, the first cutter invades the frozen soil, the frozen soil is compressed by the strength of the damage, at this time, the expression of the normal force of the cutter $F_{z1}$ is:

2 $$F_{z1} = \sigma_{c} h_{1} \pi r\sqrt {r^{2} + h_{1}{^{2}} }$$

In which:$\sigma_{c}$ is the frozen soil compressive strength (MPa), which can be determined by test or based on empirical parameters; $h_{1}$ is the depth of frozen soil intruded by the cutter in the preliminary intrusion stage (mm), and $r$ is the radius of the cutter (mm).

The rolling force $F_{y1}$ is the sum of its own gravity, the rotational force of the drum, and the inertial force, and the formula is organized as the expression:

3 $$F_{y1} = \mu Mg + ML\omega^{2} \cos \theta + \sigma_{c} h_{1} \pi r\sqrt {r^{2} + h_{1}{^{2}} } \cdot \sin^{2} \theta$$

In which: $\mu$ is the friction coefficient of frozen soil, generally taken as 0.2, $M$ is the total weight of the new vertical shaft tunneling machine (kg), $L$ is the distance from the center of the drum to the tip of the cutter (mm), and $\omega$ is the angular speed (rpm).

The lateral force $F_{x1}$ is expressed as:

4 $$F_{x1} = \frac{2}{3}\tan \theta \cdot \tau_{0} \sqrt {2rh_{1} - h_{1}{^{2}}}$$

In which: $\tau_{0}$ is the shear strength of frozen soil (MPa).

For the localized crushing stage, which is formed by two rolling intrusions of the two cutters into the frozen soil, only the forces on the second cutter need to be calculated when calculating the force situation.

The normal force $F_{z2}$ on the cutter is expressed as:

5 $$F_{z2} = F_{z1} + 2\sigma_{c} h_{2} \pi r\sqrt {r^{2} + h_{2}{^{2}} } \,$$

In which: $h_{2}$ is the depth of intrusion of cutter into frozen soil in localized crushing stage (mm).

Rolling force $F_{y2}$ The expression is:

6 $$F_{y2} = F_{y1} + 2\sigma_{c} h_{2} \pi r\sqrt {r^{2} + h_{2}{^{2}} } \cdot \sin^{2} \theta$$

The lateral force $F_{x2}$ expression is:

7 $$F_{x2} = F_{x1} + \frac{2}{3}\tan \theta \cdot \tau_{0} \sqrt {2rh_{2} - h_{2}{^{2}} }$$

For the regional breaking frozen soil stage, the second cutter rolls into the frozen soil to make the two regions pass through, and the normal force, rolling force and lateral force on the cutter are consistent with the calculation formula of the local intrusion stage.

From Eqs. (1)–(7), it can be seen that in the process of breaking frozen soil by the cutter, because the angle of the cutter intrusion is fluctuating in a range, the lateral force, rolling force and normal force curve of the cutter is a fluctuating curve with pulsation.

In the numerical simulation of high strain rate problems, where the numerical solution of a system of stress differential equations presents a number of difficulties, Von Neumann and Richtmyer added an artificial volumetric viscous q to the pressure term to blur the strong intermittency of the stress wave into a sharply varying, but continuous, situation in a rather narrow region^2^. The standard algorithm for the artificial volumetric viscosity q is:

8 $$\begin{aligned} q & = \rho l\left( {C_{0} l\left| {\dot{\varepsilon }_{kk} } \right|^{2} - C_{1} a\left| {\dot{\varepsilon }_{kk} } \right|} \right) \quad IF\dot{\varepsilon }_{kk} & 0 \hfill \\ {\text{q} } & = 0 \quad IF\dot{\varepsilon }_{kk} \ge 0 \hfill \\ \end{aligned}$$

In which: the characteristic length is $l =\sqrt[3]{{V}}$, $a$ is the local sound velocity, $\rho$ is the mass density after the failure of the frozen soil, $\left| {\dot{\varepsilon }_{kk} } \right| = \left| {\left( {\dot{\varepsilon }_{11} + \dot{\varepsilon }_{22} + \dot{\varepsilon }_{33} } \right)} \right|$ is the trace of the strain rate tensor, $C_{0}$ and $C_{1}$ is a dimensionless constant, and its default values are 1.5 and 0.006.

After introducing the artificial volume viscosity q, the stress calculation formula is:

9 $$\sigma_{ij} = S_{ij} + \left( {p + q} \right)\delta_{ij}$$

In which: *p* is the force acting on the pick to break the frozen soil, $S_{ij}$ is deviatoric stress tensor, $\delta_{ij}$ Represents the mark of Cormac.

**Figure 4:**
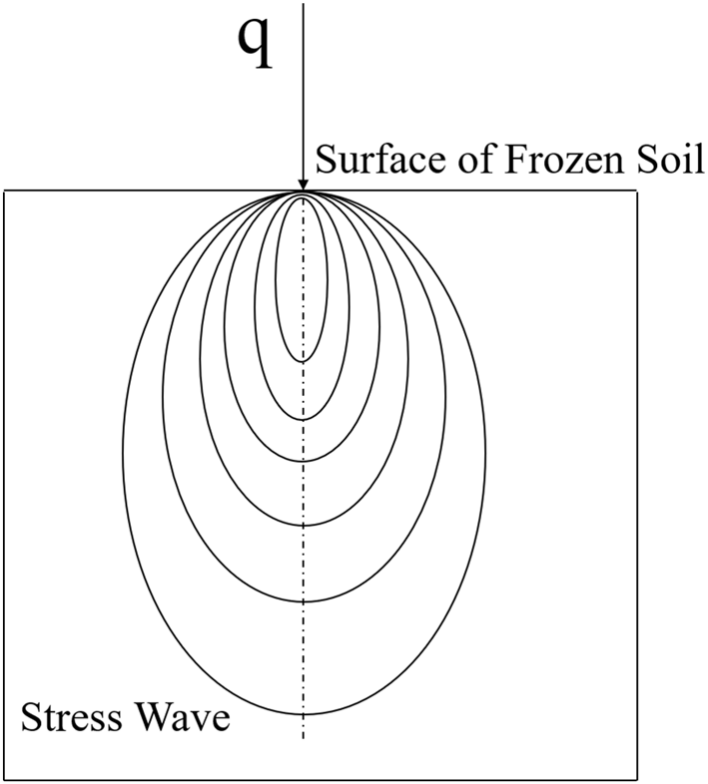
Regular internal stress distribution in frozen soil under linear loading.

The New Shaft Tunneling Machine rolling cutter in the body of the self-gravity, drum rotation force and inertia force under the joint action of the invasion of frozen soil, cutter force exceeds the bearing capacity of the frozen soil itself when the fracture, detachment from the soil, and ultimately, the frozen soil tensile, compression and shear damage. After the introduction of artificial volume viscosity in the numerical simulation calculation of stress calculation by the formula (9) can be seen, the bias stress tensor is certain^31,32^, the greater the cutter breaks the frozen soil force, the greater the value of stress, so the cutter and the initial contact area of the frozen soil stress value is the largest, and gradually decreases in all directions. As shown in Fig. 4, the normal stress inside the frozen soil is distributed in the form of stress wave spreading downward and around infinitely, and the stress distribution is basically symmetric^28^.

### Analysis of FEM-based cutter breaking frozen soil process

#### Model establishment

The software SolidWorks is used to establish the model of cutter breaking frozen soil. The diameter of the drum of the new shaft tunneling machine is 580 mm, the length of the cutter is 103 mm, and the length of the frozen soil specimen is 1500 mm, the width is 450 mm, and the height is 300 mm. In order to improve the computational efficiency, the frozen soil is divided by the segmentation line during modeling, which is convenient for local encryption during the mesh division of the frozen soil specimen.

#### Model meshing and parameter setting

The finite element software HyperMesh was used for meshing and parameterization of the cutter and frozen soil materials. Tetrahedral mesh was used for the frozen soil entity, while trihedral mesh was used for the cutter entity, with a total of 817,845 nodes and 1017,878 mesh cells. The Card Image of the cutter material was *MATL20, with a density of 7900 kg/m^3^, a modulus of elasticity of 270 GPa, and a Poisson's ratio of 0.3; the Card Image of the frozen soil material was *MATL111, and the parameters of the frozen soil material were adopted from the clay material of an underground mine^8^, and a series of test appliances were used to configure the granular-graded soils of a specific water content into specimens with the dimensions of 50 mm × 25 mm and 50 mm × 100 mm specimens using a series of test tools, and stored as frozen soil at a constant temperature under specific temperature conditions. WAW-600 axial compression testing machine was used to carry out frozen soil physical tests^33^, real-time data collection during specimen loading, and finally, based on the initial parameters of the HJC compression damage model, the dynamic damage intrinsic model of frozen soil was obtained after optimization^34–36^, and the data are shown in Table 1. In the table: *ρ* is density, *G* is shear modulus, *f′c* is static and quasi-static compressive strength, *T* is tensile strength; *A, B, C, N, S*_*Max*_ are strength parameters; *D*_*1*_, *D*_*2*_, *εf*_*min*_ are fatigue damage parameters; *P*_*c*_, *μ*_*c*_, *P*_*l*_, *μ*_*l*_, *K*_*1*_, *K*_*2*_, *K*_*3*_ are pressure parameters; *ε*_*0*_ is reference strain rate, *f*_*s*_ is failure type.

**Table 1:** Frozen soil material parameters.

$\rho$/(kg/m^3^)	$G$/GPa	$A$	$B$	$C$	$N$	$f_{c}^{\prime}$/MPa
2100	2	1.2	0.5	0.012	1.0	9
$\varepsilon_{0}$/${\text{s}}^{ - 1}$	$\varepsilon f_{min}$	$S_{max}$	$P_{c}$/MPa	$\mu_{c}$	$P_{l}$/MPa	$D_{1}$
1.0	0.01	7.0	16	0.001	80	0.04
$D_{2}$	$\mu_{l}$	$K_{1}$/GPa	$K_{2}$/GPa	$K_{3}$/GPa	$T$/MPa	$f_{s}$
1.0	0.1	85	− 171	208	0.3	0.004

**Figure 5:**
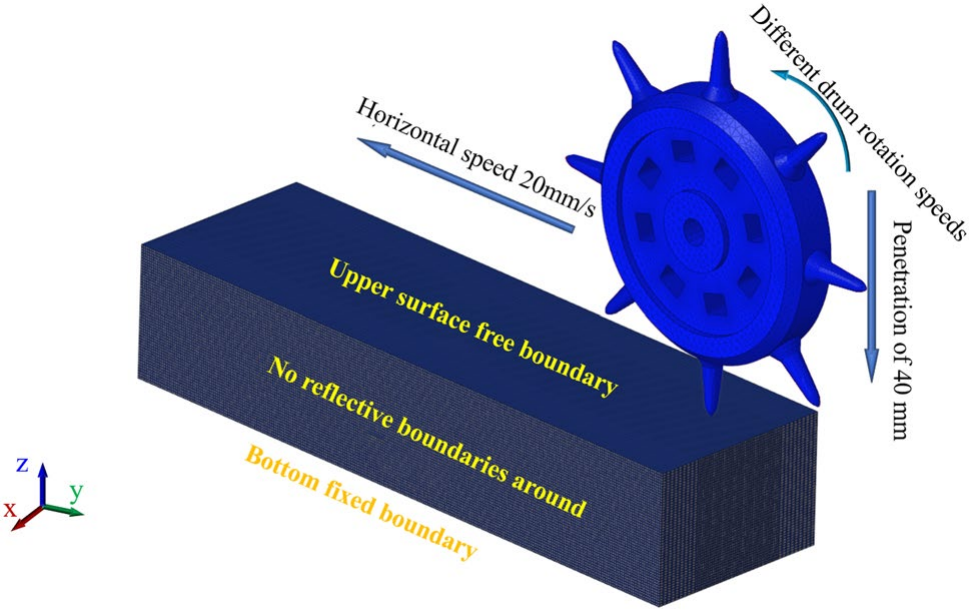
Setting of boundaries for model constraints of cutter breaking frozen soil.

In order to refine the simulation of cutter digging into frozen soil layer in the vertical dimension, Hyper Mesh software is used to set the model parameters. The calculation time of the simulation of the cutter breaking frozen soil is 0.2 s, and the time step is 7e−6s, which saves the calculation time and ensures the reasonable data at the same time. In order to realistically simulate the propagation of stress inside the frozen soil^8^, the upper surface of the frozen soil model is set as a free boundary, the left and right side faces and the front and back two faces are used as non-reflective boundaries, and the lower surface is set as a fixed boundary, not counting the influence of the self-weight of the frozen soil, as shown in Fig. 5.

After the parameter setting is completed, the .k file is output and solved using the solver LS-DYNA to derive the process of breaking frozen soil by the cutter at different drum rotation speeds.

### Field test process

In order to further verify the process of breaking frozen soil under the optimal drum rotation speed of the new shaft tunneling machine, an internal simulation test is carried out to break frozen soil with cutter under the optimal drum rotation speed based on the actual situation in the field. Due to the influence of the actual environment of the shaft and considering that the new shaft boring machine is still in the trial operation stage, the field test environment cannot fully meet the conditions of production and preservation of massive frozen soil, for this reason, the C30 concrete is used as the test object, and the mechanical properties of the specimens measured through the uniaxial compression test, Brazilian splitting test, etc. are shown in Table 2.

**Table 2:** Mechanical properties of C30 concrete.

Parameter name	Unit	Number
Density	kg/m^3^	2360.00
Elastic modulus	GPa	23.44
Compressive	MPa	30.31
Tensile strength	MPa	3.56
Cohesion	MPa	4.19
Angle of internal friction	(°)	30.00

To verify the process of cutter breaking frozen soil under the optimal drum rotation speed, the optimal drum rotation speed equivalent to the numerical simulation was used for the cutter breaking concrete test, and a high-speed video camera was used to record the process of cutter breaking concrete, and observe the degree of damage and the extent of breaking frozen soil under the optimal drum rotation speed^8^.

**Figure 6:**
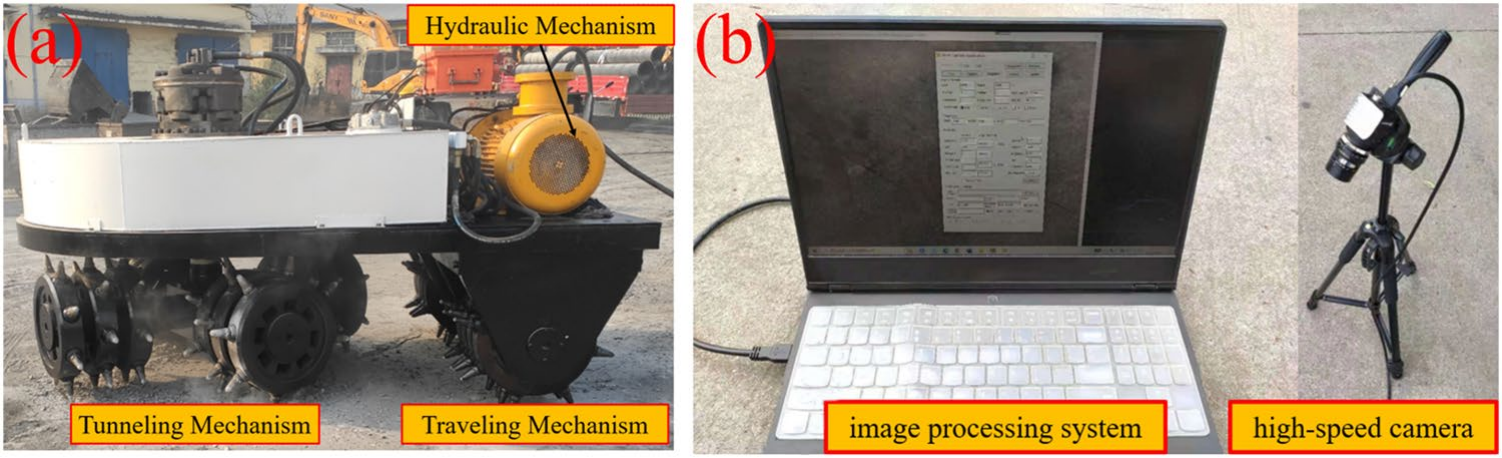
Field test setup (**a**) the new shaft tunneling machine, (**b**) high-speed camera.

In order to meet the requirement of rolling concrete crushing by the cutter, the hydraulic motor torque of the boring mechanism is 30 kN·m, as shown in Fig. 6a. In order to facilitate the recording and measurement of the rolling concrete crushing distance of the cutter as well as the crushing depth, it was recorded with the help of a Lt425 high-speed camera and a steel ruler. The Lt425 high-speed camera is a 4-megapixel USB3.0 industrial camera, which provides 2048 × 2048 pixels using 5.5 μm^2^ pixels, with a high frame rate of 90 fps as shown in Fig. 6b.

## Results and discussion

In order to determine the optimal drum rotation speed and improve the efficiency of cutter breaking frozen soil, according to engineering practice^37^, we mainly analyze the frozen soil damage condition, the change law of cutter breaking frozen soil force, frozen soil stress–strain, and energy when the penetration degree of rolling cutter is 40 mm, the horizontal movement speed of the drum is 20 mm/s, and the rotation speed is 25–40 rpm.

### The frozen soil damage condition under different drum rotation speeds

**Figure 7:**
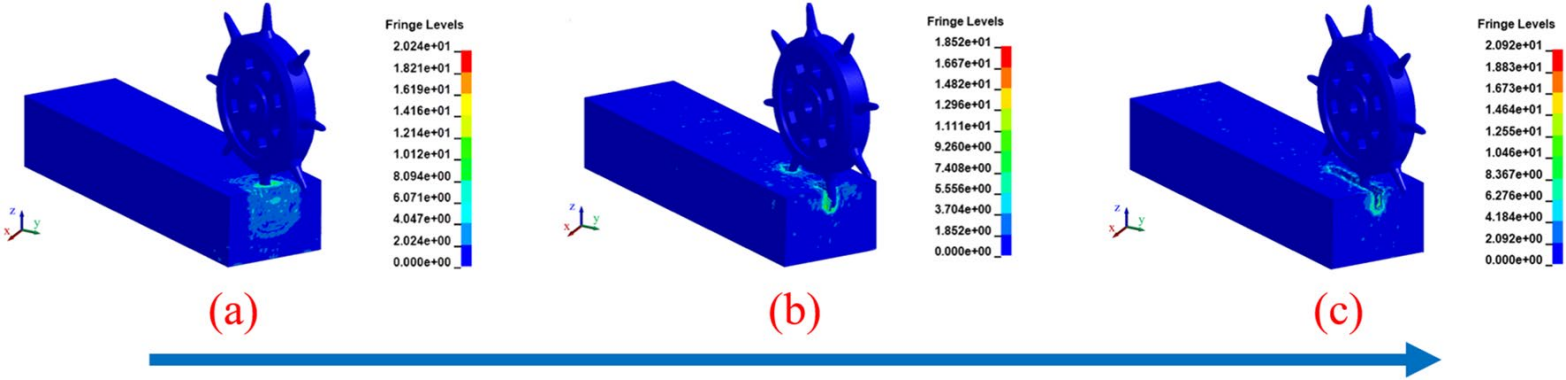
Von Mises stress diagram for rock breaking process of cutter (**a**) initial intrusion stage (**b**) localized crushing stage (**c**) regional crushing stage.

The breaking frozen soil by cutter is a continuous process, which should be analyzed from macro- and microscopic point of view to analyze the characteristics of frozen soil damage^38^. When the cutter starts to rotate and translate at a specific rotational speed and contacts the frozen soil, as shown in Fig. 7a, the surface of the frozen soil instantly produces a dark-colored patch of clouds, indicating that a stress change is generated at this moment. With the increase of penetration of the cutter, the stress increases gradually. The unit missing and small ripples are generated around the area where the frozen soil and the cutter acted, which indicates that the concentrated stress is generated when the frozen soil and the cutter are in contact, the strain rate effect occurs in the frozen soil, and the compressive, tensile and shear strengths of the frozen soil are increased. Cracking and breaking frozen soil in the area of interaction with the cutter, and there is a depth of depression damage.

With the successive rolling of the cutter, the other cutter started to contact the frozen soil, as shown in Fig. 7b. The internal section of frozen soil was impacted by two cutters, which produced different depths of unit missing, and there were phenomena such as dark-colored areas extending downward. It indicates that at this time, the internal stress range of frozen soil is enlarged and the degree of breaking frozen soil is aggravated, which produces more small cracks, and numerous micro-cracks gradually merge into the main cracks. Due to the high rotating speed of the drum, the frozen soil in the process of brittle damage to plastic damage, the internal integrity did not reach the balance and once again subjected to the high-speed rolling impact of the cutter, the overall structure of the frozen soil did not reach the transient equilibrium and once again occurred in the depression damage, so that the frozen soil off the broken frozen soil to achieve the purpose of excavation of the frozen soil.

When the frozen soil is subjected to continuous rolling contact by multiple cutters, the frozen soil shows a area of color change, more units are missing, the internal cross-section is densely rippled and there are a lot of splashed broken unit blocks, indicating that the frozen soil damage is gradually transformed from the plastic damage stage to the plastic compaction and deformation stage, and tearing and breaking, crack expansion and depression compaction phenomena occur in the parts of the frozen soil that are in contact with the cutter, as shown in Fig. 7c. As the frozen soil has been disturbed and damaged, the stress value gradually decreases and the strain convergence phenomenon occurs. This completes one round of frozen soil breaking process.

It can be seen that the frozen soil in different stages of damage, the role of the region at the color change are more significant, indicating that the cutter will accelerate the frozen soil stress fragmentation, frozen soil model damage, the internal presentation of a number of transverse cracks and vertical cracks, with the cracks through each other, resulting in a larger crack, the unit failure deleted, the depth of the frozen soil depressions and the damage to the area increased, the destruction of the effect is significant.

**Figure 8:**
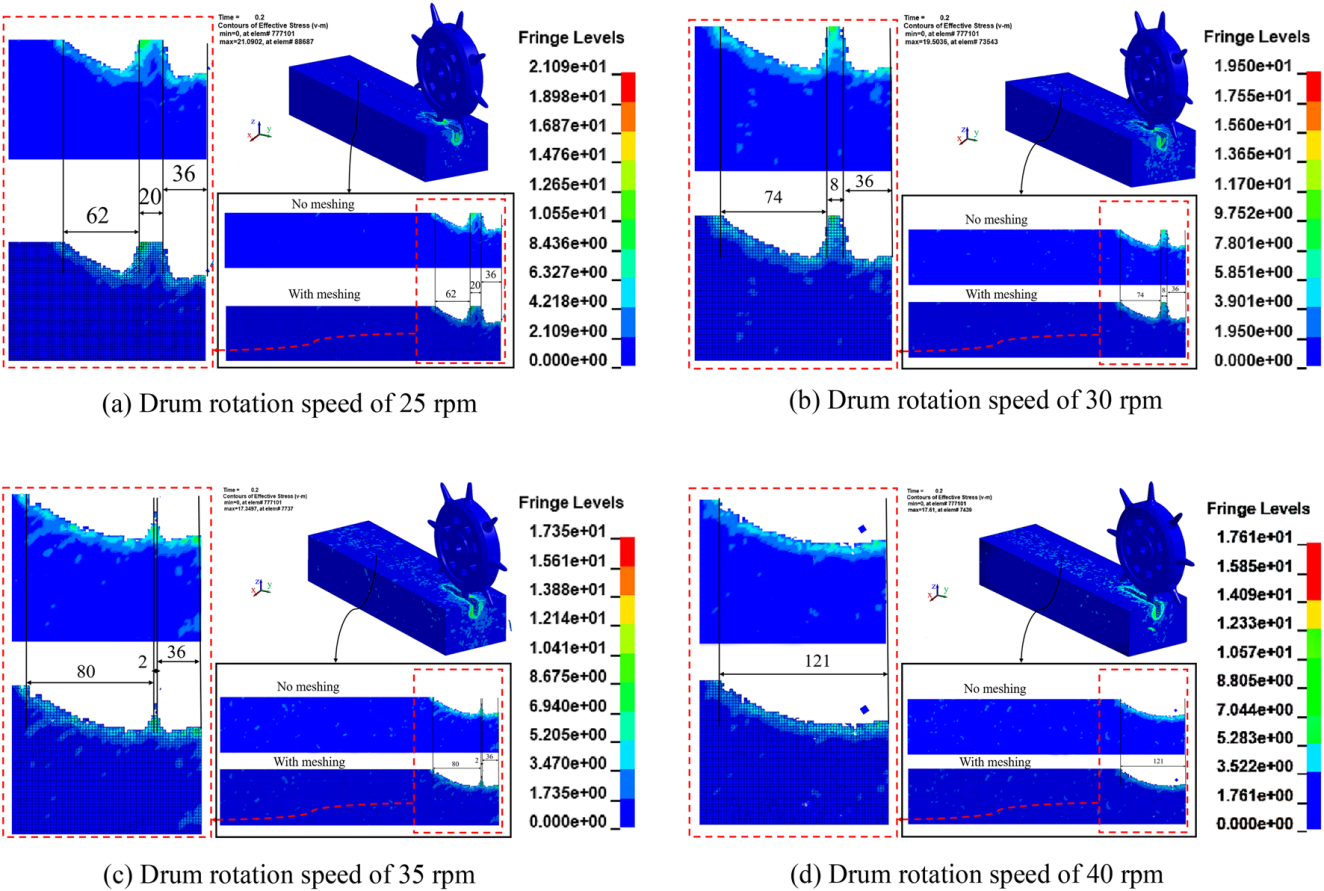
Frozen soil damage condition at the moment of 0.2 s under different drum rotation speeds.

In 0.2 s, when the drum rotation speed was set to 25 rpm, the color area appeared in the frozen soil internal cross-section and was accompanied by missing units and splashing phenomenon, indicating that the frozen soil produced concentrated stress under the action of high-speed rotation of the cutter, which caused cracks in the contact area, and depression damage and fragmentation^39^, but the two sections of the broken frozen soil were not connected, and the final horizontal breaking distance was 98 mm, see Fig. 8a; as shown in Fig. 8b, when the drum rotating speed is 30 rpm, the frozen soil cross-section has a color concentration and color change area, which indicates that by increasing the rolling speed of the cutter, the internal structure of the frozen soil has a region of high stress change and rapidly expands to the surrounding area, and at the same time accelerates the detachment of the frozen soil in this region. It is to see that the two crushed areas are still not through, compared with 25 rpm, 12 mm shorter, and the final horizontal crushing distance is 110 mm.

When the roller rotation speed is 35 rpm, the whole internal section of frozen soil has color change, continuous corrugation on the section extends to the bottom of the frozen soil section, and there are still untimely dislodged unit blocks in the missing unit area, which indicates that the destructive stress of the frozen soil is greater than the maximum bearing stress of the frozen soil itself, the destructive intensity is large, the crack expands to be the main crack, and there are more fragments produced, and there is still a distance of 2 mm between two sections of the crushed area that is not through, and the final The horizontal breaking distance is 118 mm. as shown in Fig. 8c; as shown in Fig. 8d, when the drum rotating speed is 40 rpm, the color area change of the internal section of frozen soil decreases, but the dense corrugation increases significantly, and the main crack, which indicates that the dense structure of the frozen soil has been disturbed and damaged, and when the damaged frozen soil contacts with the rolling cutter again, the change of the internal stress decreases, and the frozen soil is damaged. The frozen soil damage reached the stage of regional breaking, and the final horizontal breaking distance was 121 mm.

### Variation of the combined force of the cutter at different drum rotation speeds

**Figure 9:**
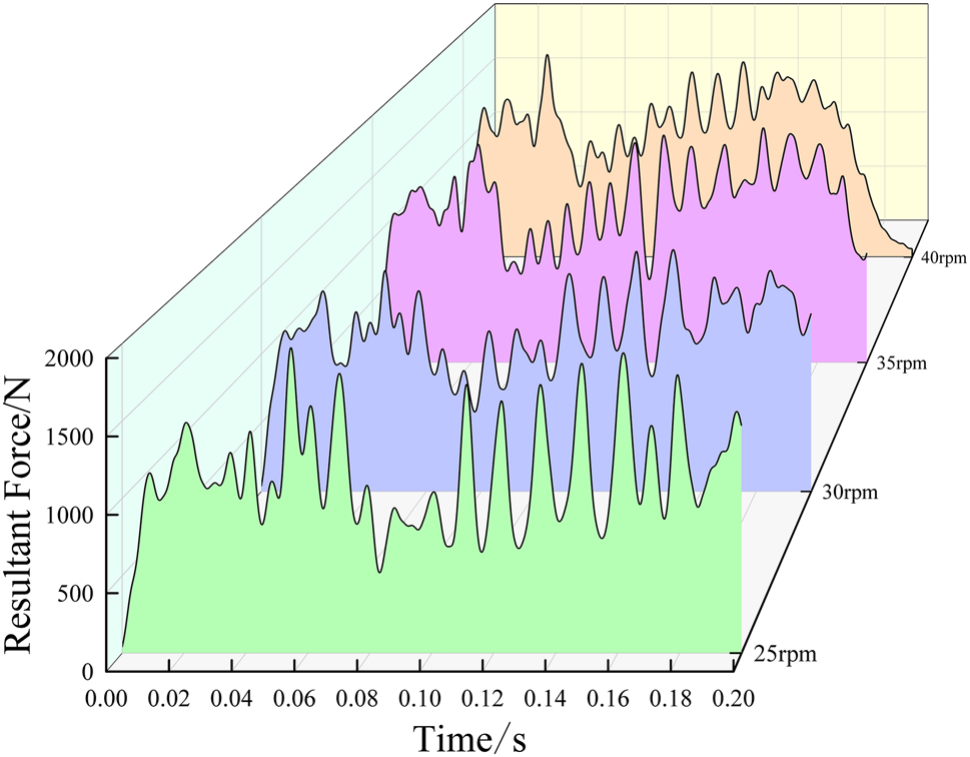
Cutter resultant force curves under different drum rotation speeds.

Increase the drum rotation speed, the cutter acting on the frozen soil combined force is different. When the initial intrusion of the cutter into the frozen soil, the force reaches a peak, with the forward rotation of the drum, the cutter again rolling to destroy the frozen soil produces a greater force, after that, due to the frozen soil has been interfered with the destruction, the force gradually decreases, a unit of dislodging is completed, the force of the cutter will be reduced to zero or tend to be zero, due to the continuous formation of dense nuclei and dislodging of the size and time cannot be determined^40^, so that the combined force curve is an irregular fluctuating change curve, as shown in Fig. 9, which is consistent with the theory of force analysis of cutter breaking frozen soil.

**Figure 10:**
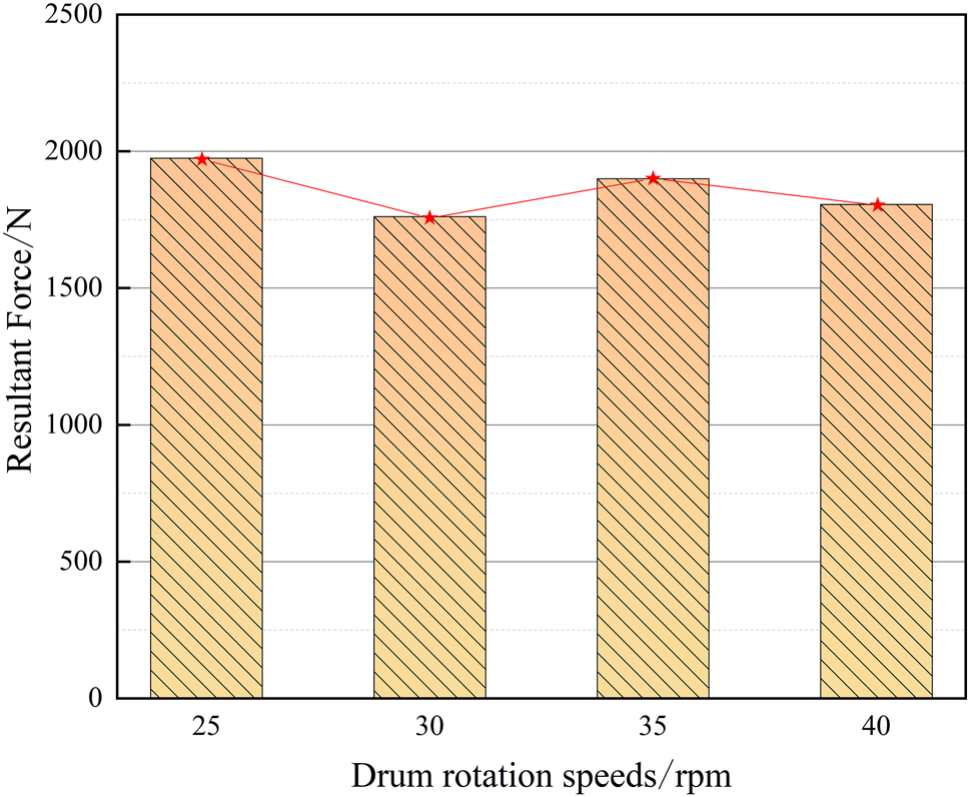
Cutter resultant force peak curves under different drum rotation speeds.

The cutter force decreases the fastest when the drum rotation speed is 40 rpm, which indicates that the frozen soil has been completely destroyed without the need for a larger force, and the faster the decrease, the more serious the destruction of frozen soil is proved to be. As can be seen from Fig. 10, the corresponding peak force is 1975, 1761, 1900 and 1806 N when the drum rotation speed is 25, 30, 35 and40 rpm, and the comparative analysis shows that the cutter have a wide range of cracks, breaking soil and depression damage under different drum rotation speeds, and the same penetration degree. Under the same penetration, the rotating speed of the drum is 30 rpm, the required force is the smallest, and it meets the requirements of frozen soil damage.

### Stress–strain changes of frozen soil under different drum rotation speeds

**Figure 11:**
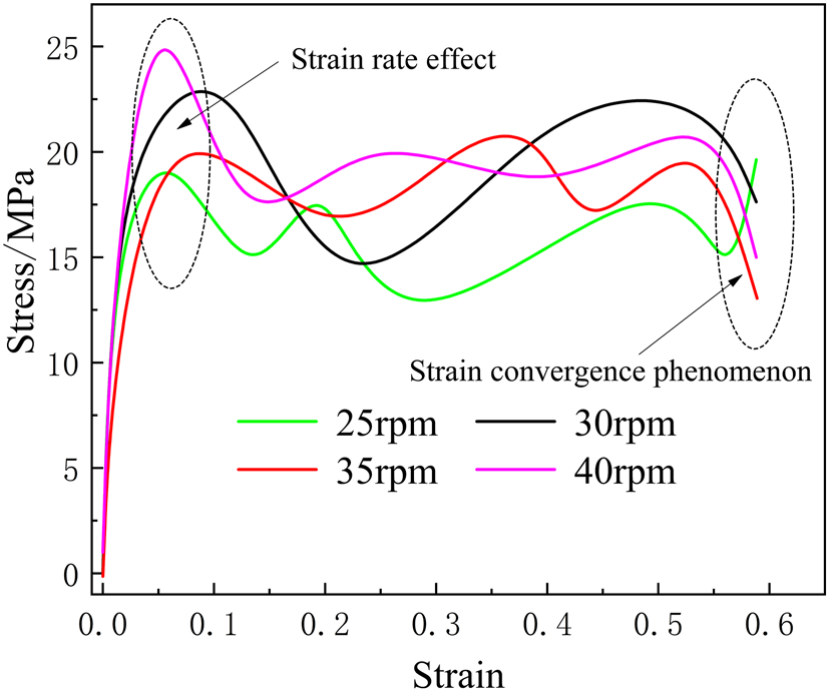
Stress–strain curve of frozen soil under different drum rotation speeds.

In addition, in the study of frozen soil mechanical damage, the strain convergence phenomenon is an basis for judging frozen soil damage, where the drum rotation speed is one of the factors in generating this phenomenon^41,42^. The results show that the frozen soil stress–strain curves differ significantly under different drum rotation speeds but do not affect the appearance of the strain rate effect; the instantaneous strength of frozen soil increases when it is exposed to a impact force, and the peak strain change trend is consistent and is accompanied by the appearance of the strain convergence phenomenon, and the frozen soil is damaged^8^. The analysis shows that the peak stress gradually increases as the drum rotation speed increases, and the strain end values of the frozen soil stress–strain curve are consistent regardless of the change in drum rotation speed, as shown in Fig. 11.

As shown in Fig. 11, when the high-speed rotation of the cutter destroys the frozen soil, the internal stress of the frozen soil reaches the peak value instantly, while the strain change of the frozen soil is small, and the strain rate effect unique to frozen soil is reflected. The instantaneous strength of frozen soil is great when contacting the cutter, and the force of cutter is not enough to destroy the frozen soil, but when the cutter accelerates the rotation, the strain rate effect of frozen soil disappears, the shear, tensile and compressive strengths decrease, and the local structure of frozen soil enters into the plastic yielding stage. When the drum rotating speed is 25 rpm, the maximum stress of rolling cutter breaking the frozen soil is 22.3 MPa, the strain change in a short period of time is relatively flat, and the destructive ability of frozen soil is weak. At 30 rpm, the maximum stress of the rolling cutter to break the frozen soil reaches 23.2 MPa, and the strain change is large, which is strong for the destruction of frozen soil. The maximum stress of the rolling cutter breaking the frozen soil is 23.7 MPa and 24.8 MPa when the roller rotating speed is 35 rpm and 40 rpm, respectively, and the degree of frozen soil damage is more serious.

### Variation of specific energy under different drum rotation speeds

**Figure 12:**
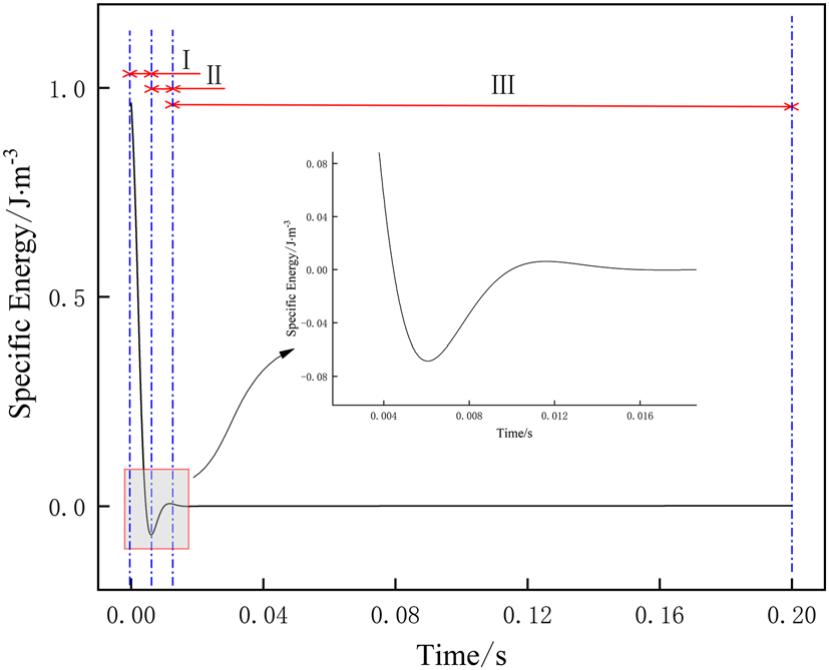
Variation curve of specific energy under different drum rotation speeds (the specific energy curve is divided into three sections according to the trend of specific energy curve and the local enlargement).

Specific energy is an index to reflect the efficiency of breaking frozen soil by cutter, i.e. the smaller the specific energy is, the lower the energy required to break frozen soil per unit volume of cutter is, and the higher the efficiency of breaking frozen soil is and it is more economical^24^. When the first contact of the cutter with frozen soil, the energy required for breaking frozen soil per unit of frozen soil is the largest, due to the disturbance damage of frozen soil after the crushing stage, so it does not need the cutter to break it with a larger force, and the energy required for breaking frozen soil gradually decreases. Finally, with the completion of breaking frozen soil by the cutter, the energy required for breaking frozen soil by the cutter tends to stabilize, and the specific energy curves under different drum rotation speeds are shown in Fig. 12.

The results show that the trends of specific energy change of the cutter breaking frozen soil are similar at different rotational speeds, which can be roughly divided into three stages: (I) decreasing stage, (II) increasing stage and (III) horizontal stage. When the frozen soil is initially invaded by the first cutter, due to the special nature of frozen soil, the cutter needs to consume more energy, and after the frozen soil is disturbed and destroyed, it doesn't need to consume too much energy, so the specific energy curve starts to decline, under the same conditions, with the increase of time, the specific energy of breaking frozen soil gradually decreases, which means that the efficiency of breaking frozen soil is gradually improved, so the faster the trend of the specific energy decreases under the different drum rotation speeds, which proves that the drum rotation speed is optimal; with the intrusion of the second rolling cutter, part of the frozen soil is broken twice, the energy consumption increases, and the specific energy curve rises; finally the frozen soil is broken and dislodging occurs, and the energy consumption tends to stabilize.

**Figure 13:**
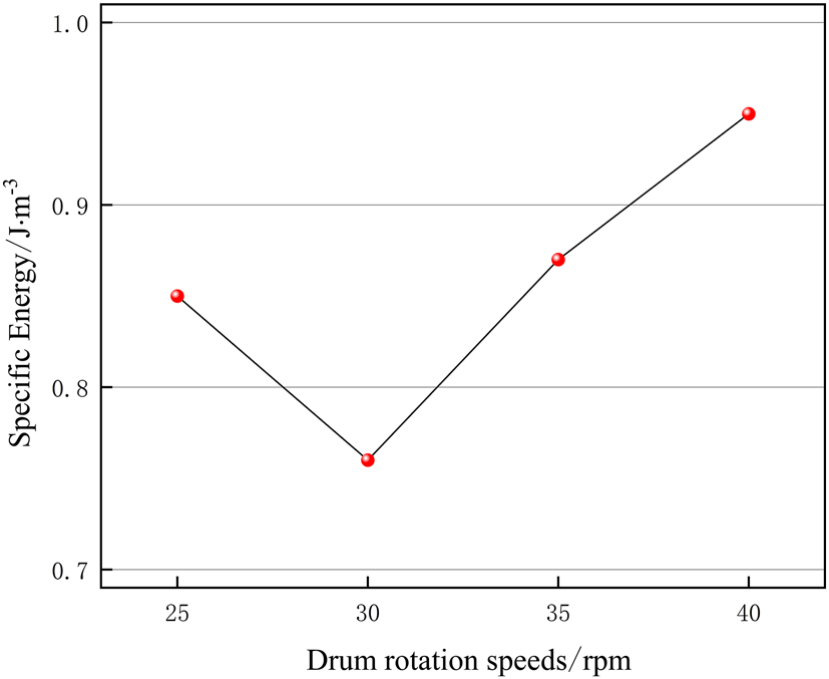
Specific energy curves under different drum rotation speeds.

As shown in Fig. 13, the specific energy decreases rapidly when the drum rotation speed is 30 rpm, which proves that the efficiency of breaking frozen soil is high, and with the increase of drum rotation speed, the trend of specific energy decreasing slows down, and the efficiency of breaking frozen soil decreases.

With the increase in drum rotation speed, the specific energy did not become smaller because: (1) the recompacting of the crushing area under the cutter, resulting in the generation of smaller particles of broken soil chips, the secondary breaking frozen soil consumes additional energy, so that the specific energy of crushing increases; (2) the cutter force is not enough to produce a number of broken soil pieces, the energy consumption has not been reduced, and thus the specific energy of crushing did not become smaller due to the increase in the force.

As a conclusion, when the drum rotating speed is 25 rpm, the total energy is lower, but the specific energy and the cutter force are both maximum and the ability to break frozen soil is weak; when the drum rotating speed is 30 rpm, the specific energy and the total energy are both minimum, and the ability to break frozen soil is strong; when the drum rotating speed is 35 rpm, the specific energy is low and the cutter force to break frozen soil is strong, but the total energy and the force of cutter are relatively higher; when the drum rotating speed is 40 rpm, the cutter force is minimum and the ability to break frozen soil is strong, but the specific energy and the total energy are relatively high. Compared with the maximum rotational speed of the drum, the difference in the ability of the cutter to break the frozen soil when the rotational speed is 30 rpm is not significant, and the specific energy is the smallest. Therefore, the overall data analysis concludes that the drum rotation speed of 30 rpm is optimal, and the efficiency of the cutter to break the frozen soil is high and the destruction of frozen soil is obvious.

### Analysis of the results of field tests

The new shaft tunneling machine cutter breaking concrete is a gradual process, which can be divided into three stages: local area contact, local area intrusion, and area crushing^16,43^. From cracks sprouting, expanding and intersecting, to the formation of debris and stripping from the concrete body. The presence of defects such as microcracks, pores and weak surfaces within the concrete makes its physico-mechanical properties, showing non-homogeneous mechanical properties, which strongly affect the concrete rolling crushing mechanism^38,39^.

**Figure 14:**
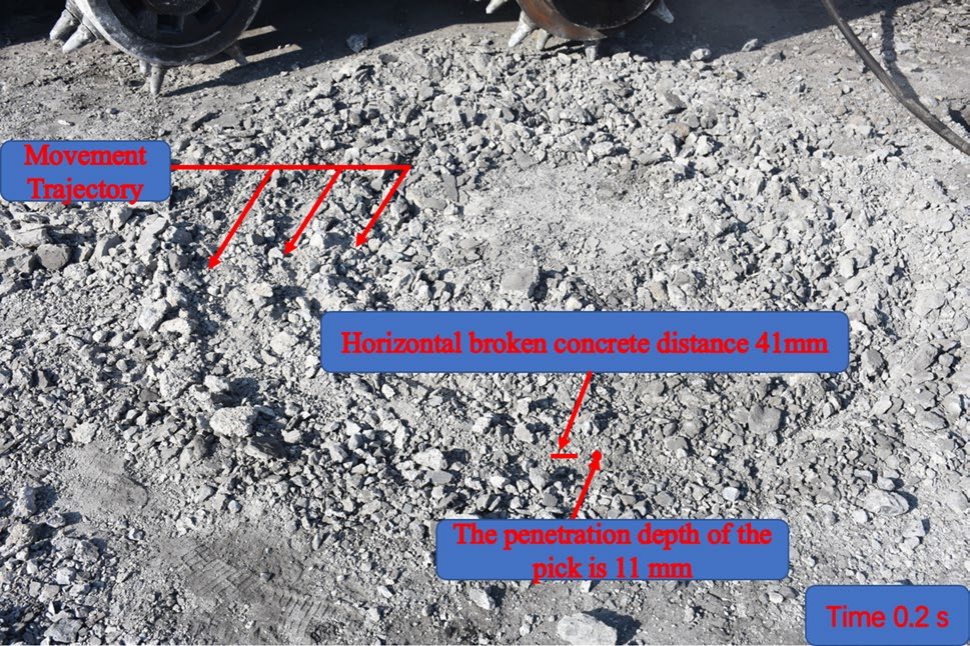
Result of concrete breaking by new shaft tunneling machine under optimum drum rotation speed.

Under the optimal drum rotation speed, the rolling cutter and concrete local area contact stage, the concrete surface and cutter contact point near the area of stress concentration, the concrete instantly produce a number of micro-cracks; with the continuous intrusion of the cutter, the micro-cracks quickly extended to large cracks, resulting in large cracks gradually intersected, the formation of concrete debris and detachment from the body; with the high-speed rotation of the drum, the number of concrete debris and the small size of the concrete debris. The damage is more serious. This is due to the cutter in the drum continuous rapid rotation of the damaged concrete and has fallen off the broken pieces of further rolling broken, so that the concrete depression damage is significant, the final damage is shown in Fig. 14.

To further validate the test results, the data from numerical simulation and field frozen soil breaking test were compared and analyzed, and the differential rate of rolling frozen soil breaking distance of the cutter and the incremental rate of the depth of intrusion of the cutter were calculated by Eqs. (10) and (11).

10 $$\delta = \frac{{l_{0} - l_{1} }}{{l_{1} }}$$

In which: *l*_0_ is the rolling distance of cutter to break frozen soil for test value (mm), *l*_1_ is the simulated value of the rolling distance of the cutter to break frozen soil (mm), and *δ* is the rate of difference in the rolling distance of the cutter to break frozen soil (%).

11 $$\Delta = \frac{{13 - H_{i} }}{{H_{i} }}$$

In which:*Δ* is the incremental rate of depth of cutter intrusion (%), *H*_i_ is the depth of cutter intrusion corresponding to the optimal rotation speed of the drum (mm).

Substituting 115 mm of horizontal frozen soil breaking distance of the cutter in the field test and 110 mm of horizontal frozen soil breaking distance in the simulation within 0.2 s into Eq. (10), and 11 mm of depth of intrusion of the cutter into Eq. (11), the difference rate of rolling frozen soil breaking distance of the cutter is 4.5%, and the incremental rate of depth of intrusion of the cutter is 18.2%. It is proved that the data obtained from numerical simulation are closer to the test situation, which verifies the reliability of the simulation results.

Through the field test, it can be seen that the frozen soil produces a number of microcracks under the optimal drum rotation speed, and with the intersection of microcracks, the fragments are formed and detached from the soil body, and due to the high-speed rotation of the drum so that the dislodged soil is damaged again, and the frozen soil crushed in numerous but tiny sizes.. Consistent with the numerical simulation results, it proves that the frozen soil excavation under the optimal drum rotation speed is efficient and effective.

## Conclusion

1. Under different drum rotation speeds, the form of frozen soil damage affects the characteristics of its stress–strain curve, and then the strain rate effect and strain convergence phenomenon appear. Under the optimal drum rotation speed, the cutter intrudes into the frozen soil under the joint action of body gravity, drum rotation force and inertia force, and when the bearing capacity of the frozen soil itself is lower than the force of the cutter, there is a fragmentation and detachment from the soil, and the frozen soil undergoes tensile, compressive and shear damage.
2. Under the optimal drum rotation speed, the frozen soil stress breaking frozen soil speed accelerates, generates larger cracks, increases the depression depth and damage area, and the effect of frozen soil destruction is significant. Within a range of drum rotation speed, with the increase of drum rotation speed, the main cracks of frozen soil increase, the area of frozen soil depression and the degree of damage increases, and the cutter breaks the frozen soil at a drum rotation speed of 30 rpm with the lowest force and peak specific energy, which increases the efficiency of the cutter to break the frozen soil by 10.8% compared to the drum rotation speed of 40 rpm.
3. Combined with the field test of breaking frozen soil under the optimal drum rotation speed of the new vertical shaft boring machine cutter, the dynamic damage mechanism of frozen soil is further explored, and it is found that a number of microcracks are generated in the frozen soil under the optimal drum rotation speed, and with the intersection of the microcracks, the fragments are formed and detached from the soil body, and the difference rate of the frozen soil after damage is obtained δ as 4.5% and the increment rate Δ is 18.2%, which proves the high consistency between the numerical model and the field test data (Supplementary Information).

### Supplementary Information

Supplementary Information.

## Data Availability

The datasets generated during and/or analyzed during the current study are available from the corresponding author on reasonable request.
